# Rapid Weight Loss of Up to Five Percent of the Body Mass in Less Than 7 Days Does Not Affect Physical Performance in Official Olympic Combat Athletes With Weight Classes: A Systematic Review With Meta-Analysis

**DOI:** 10.3389/fphys.2022.830229

**Published:** 2022-04-12

**Authors:** Clóvis De Albuquerque Mauricio, Pablo Merino, Rodrigo Merlo, José Jairo Narrea Vargas, Juan Ángel Rodríguez Chávez, Diego Valenzuela Pérez, Esteban Ariel Aedo-Muñoz, Maamer Slimani, Ciro José Brito, Nicola Luigi Bragazzi, Bianca Miarka

**Affiliations:** ^1^Department of Physical Education, Laboratory of Psychophysiology and Performance in Sports and Combats, Federal University of Rio de Janeiro, Rio de Janeiro, Brazil; ^2^Núcleo de Investigación en Ciencias de la Motricidad Humana, Universidad Adventista de Chile, Chillán, Chile; ^3^Universidad Norbert Wiener, Lima, Peru; ^4^Escuela de Kinesiología, Facultad de Salud, Magister en Ciencias la Actividad Física y Deportes Aplicadas al Entrenamiento Rehabilitación y Reintegro Deportivo, Universidad Santo Tomás, Saniago, Chile; ^5^Escuela de Ciencias de la Actividad Física, el Deporte y la Salud, Universidad de Santiago de Chile, Santiago, Chile; ^6^Department of Neuroscience, Rehabilitation, Ophthalmology, Genetics, Maternal and Child Health (DINOGMI), Section of Psychiatry, University of Genoa, Genoa, Italy; ^7^Department of Physical Education, Federal University of Juiz de Fora, Governador Valadares, Brazil; ^8^Laboratory for Industrial and Applied Mathematics, Department of Mathematics and Statistics, York University, Toronto, ON, Canada

**Keywords:** rapid weight loss, diet, martial arts, dehydration, ergogenic aids

## Abstract

Given the relevance of the effects that weight loss can generate on the physical performance in athletes, this study performed a systematic review with meta-analysis of the published literature on rapid weight loss (RWL) and examined its impact on the physical performance in Official Olympic combat sports athletes. The “Preferred Reporting Items for Systematic Reviews and Meta-Analysis” (PRISMA) guidelines were followed to ensure an ethical and complete reporting of the findings. PubMed, SPORT Discus, and EBSCO were the electronic databases explored for article retrieval and selection. The following string was applied: “RWL” OR “weight loss” OR “weight reduction” AND “judo” OR “wrestling” or “taekwondo” or “boxing” AND “performance.” Based on the quality analysis, conducted according to the “Tool for the assessment of study quality and reporting in exercise training studies” (TESTEX), ten articles achieved a score >6 points. The meta-analysis showed a significant difference in pre- vs. post-weight loss (*p* = 0.003) and no effects in pre- vs. post-power and strength performance analysis (*p* > 0.05 for both results). Based on our systematic review and meta-analysis of the literature, RWL up to ≤5% of the body mass in less than 7 days does not influence performance outcomes in Official Olympic combat athletes with weight classes, considering the strength and power measures.

## Introduction

Combat sports, such as judo, kickboxing, boxing, wrestling, Brazilian jiu-jitsu, taekwondo, and karate, can be defined as sports in which two individuals engage in a one-on-one contest. The tournaments are disputed based on weight divisions (Artioli et al., 2010a; Lakicevic et al., 2020), to avoid possible injuries that may be caused by dissimilarities in strength due to a significant difference in the body mass (Reale et al., 2017). However, many athletes may wish to achieve a competitive advantage over their opponents, by using, for instance, rapid weight loss (RWL) strategies. In this sense, athletes can use nutritional (Barbas et al., 2011; Rhyu and Cho, 2014; Motevalli et al., 2015; Brandt et al., 2018; Do Nascimento et al., 2020) or physical strategies (Hall and Lane, 2001; Timpmann et al., 2012; Rhyu and Cho, 2014; Zubac et al., 2020) to decrease their body mass, including skipping meals, increasing physical exercise, restricting fluid intake, training in rubber suits, and using sauna baths, as some of the methods practiced to reduce body mass before competitions (Brito et al., 2012; Berkovich et al., 2016).

Preceding reports indicated an RWL prevalence in the range of 70–90% (Steen and Brownell, 1990; Artioli et al., 2010a; Berkovich et al., 2016; Santos et al., 2016) and 40–60% in Olympic combat sports athletes, such as wrestlers, taekwondo, boxers, and judo athletes (Kiningham and Gorenflo, 2001; Kazemi et al., 2005; Kordi et al., 2011; Viveiros et al., 2015). In a controlled setting, there is evidence that those competing in the lighter weight divisions achieve more significant relative weight losses than those in heavier weight categories (Horswill et al., 1994; Wroble and Moxley, 1998), indicating that many athletes intend to compete in the lightest weight category possible. Despite this, the magnitude of RWL does not seem to be an important factor for winning in combat sports, while the risk of weight regain (RWG) might be the decisive factor, especially for grappling sports (Alderman et al., 2004; Reale et al., 2016; Zubac et al., 2018; Coswig et al., 2019; Faro et al., 2021; Pélissier et al., 2022). In addition, few studies showed the magnitude of the effect of RWL, called “weight cutting” and “making weight,” on athletes’ power or strength. This analysis can help guide professionals who want to improve the athlete’s performance and the probability of success using RWL without losing physical performance during competition.

The RWL next to the combat tournaments is omnipresent (Drid et al., 2021), with this behavioral pattern seeming to be highly practiced by athletes (Tarnopolsky et al., 1996; Hall and Lane, 2001; Koral and Dosseville, 2009; Artioli et al., 2010a; Timpmann et al., 2012; Lopes-Silva et al., 2014; Motevalli et al., 2015; Zubac et al., 2020). A few investigations assessed the relationship between RWL and competitive performance during real competitions (Horswill et al., 1994; Wroble and Moxley, 1998). Although competitive success is multifactorial, examining the associations between RWL, strength, and power, as provided by these investigations, could be insightful and help distinguish the impact of RWL on physical performance, disentangling it from other variables. In spite of conflicting evidence, most studies showed that RWL could not decrease either aerobic or anaerobic performance (Finn et al., 2004; Artioli et al., 2010a; Lopes-Silva et al., 2014; Rhyu and Cho, 2014).

Although aerobic performance has been attributed to dehydration, decreased plasma volume, hydroelectrolytic disturbances, increased heart rate, impaired thermoregulation, and muscle glycogen depletion (Fogelholm, 1994), decreased anaerobic performance was related to reduced buffering capacity, glycogen depletion, and hydroelectrolytic disturbances (Fogelholm et al., 1993). Some studies have shown that athletes who lose more weight during the hours before combat events have a higher chance of success during the bout (Artioli et al., 2010b; Rhyu and Cho, 2014). Consequently, athletes choose to be in the upper spectrum of their specific weight division to gain a physical advantage over lighter opponents (Reljic et al., 2013; Berkovich et al., 2016). Due to weight loss and regain patterns, combat sports athletes can be considered “weight cyclers” (Artioli et al., 2016). On a broader scale, athletes generally shed approximately 2–10% of their weight before each competition, primarily 2–3 days before the weigh-in (Artioli et al., 2016). The authors demonstrated relationships between the pattern of RWL practices, the potential increased risk of subsequent weight gain in weight-cycling athletes, and the need to better understand RWL (Coswig et al., 2019; Pélissier et al., 2022). These behavioral practices can be associated with poor performance outcomes, in addition to harmful side effects for fighters, which is the reason why different authors do not recommend its use (Hall and Lane, 2001; Artioli et al., 2016).

Moreover, maximal strength and power seem not to be acutely affected by RWL (Webster et al., 1990), although chronic weight cycling reduces strength gain during a season (Roemmich and Sinning, 1996). In addition, RWL is associated with acute kidney damage in combat sports athletes (Lakicevic et al., 2020); it seems that elevated biomarkers of kidney function can be primarily attributed to intentional dehydration, which is the culprit of RWL (Lakicevic et al., 2020). It is essential to highlight that the anaerobic performance is commonly observed when competitors have no opportunity to refeed and rehydrate after weigh-in (Hickner et al., 1991; McMurray et al., 1991). However, in some combat sports competitions, weigh-ins are followed by a period during which fighters may have the chance to recover from the weight loss. Although this period may vary from a few hours to more than 1 day, it is very likely that within 3–4 h, athletes could recover their anaerobic performance from pre-weight loss values (Artioli et al., 2010a). Findings are not consensual about whether and how weight cycling may lead athletes to develop physiological adaptations that could help (or not) them preserve performance after weight loss (Artioli et al., 2010b; Mendes et al., 2013). Hence, when followed by a relatively short recovery period, RWL could have an impact on anaerobic performance. However, at present, there is less direct evidence supporting these hypotheses, and systematic reviews and meta-analyses are needed to verify the effect size of RWL in a controlled setting performance. Therefore, this study aimed to realize a systematic review and meta-analysis of the published literature on RWL and Official Olympic combat athletes’ physical performance.

## Materials and Methods

### Literature Search

The “Preferred Reporting Items for Systematic Reviews and Meta-Analysis” (PRISMA) guidelines were followed for conducting a systematic review (Page et al., 2021) to ensure a transparent and complete reporting of the findings. The Mendeley software (Mendeley, United Kingdom) was used to organize the content acquired by the article searches. PubMed, SPORT Discus, and EBSCO were the electronic databases explored for article collection. The following terms were applied: “RWL” OR “weight loss” OR “weight reduction” AND “combat sports” OR “judo” OR “wrestling” or “taekwondo” or “boxing” AND “performance.”

**FIGURE 1:**
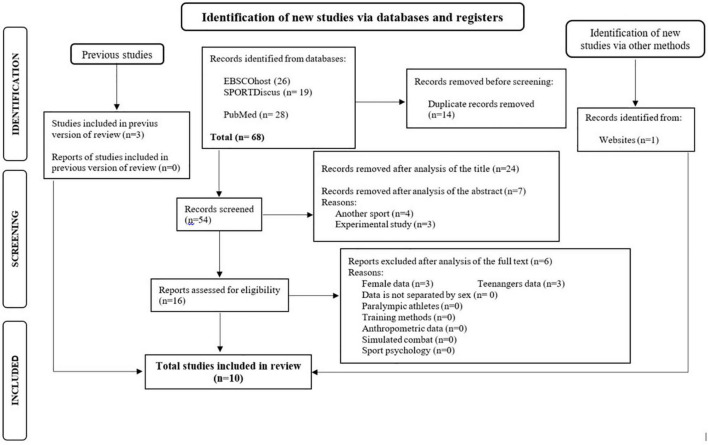
Prisma flow diagram for study selection.

The article screening was carried out three times: title, abstract, and full-text reading. If any disputes occurred between the two investigators, a third researcher reconsidered the current process independently and discussed the decision with the other investigators. Notably, the investigators were not blinded to the manuscripts, study title, authors, or associated institutions during the selection process. The screening processes have been summarized *via* the PRISMA flow diagram as shown in Figure 1.

### Inclusion and Exclusion Criteria

Only original articles written in English and published in peer-reviewed journals were considered for inclusion within this review. The date limit for the publication period was set from the year 1996 to May 2021. Reviews, meta-analyses, abstracts, citations, scientific conference abstracts, opinion pieces, books, book reviews, statements, letters, editorials, non-peer-reviewed journal articles, and commentaries were excluded.

### Type of Participants

Any combat athlete was eligible to be included in the review. Eligible articles had to be conducted in Official Olympic combats with weight classes: judo, wrestling, taekwondo, or boxing athletes, practicing an RWL method. The participant’s extent of weight loss had to be ∼5% of their body weight, and only male participants >18 years of age were selected (Khodaee et al., 2015; Sagayama et al., 2019; Lakicevic et al., 2020; Castor-Praga et al., 2021). There was no upper age limit for the participants. In addition, weight loss had to be achieved up to 10 days before the weigh-in session. If a particular study involved illegal substances to elevate the magnitude of weight loss, it was still included for further analysis. Both qualitative and quantitative articles were taken into consideration.

### Data Extraction

Critical information about included studies was extracted and presented through tables (Microsoft Word 2016, Microsoft, WA, United States), while a narrative description was performed to analyze the included literature on the topic. Certain specifics about a particular study that would expand beyond tabular explanation have been thoroughly described narratively in the “Results” section. Retrieved data acquired from included articles dealt with the influence of RWL on performance parameters of athletes.

### Quality Assessment

The purpose of the quality assessment was to detect the risk of bias, and it was carried out through the “Tool for the assessment of study quality and reporting in exercise training studies” (TESTEX) checklist (Smart et al., 2015). After the assessment, the studies were then separated into groups and labeled as “adequate quality” (score ≥7 points) or “poor quality” (score <7 points). Moreover, studies with poor quality were not included. Two authors (J-N and C-A) performed this process independently from one another, and a third author (P-M-M) acted as a referee for any doubtful case.

### Meta-Analysis

All data were combined using the Review Manager 5.3 program.^1^ The variables were sports performance indices of reaction time and decision-making measures. Our research used continuous data of mean (M), standard deviation (SD), and the number of participants (*n*). We calculated the effect size (difference between standardized means) with 95% confidence intervals (CIs) for each study, defined as the difference in mean changes after RWL (pre vs. post). The effect size was interpreted using value cutoffs of 0.2 > small, 0.5 > moderate, and 0.8 > large effect. Heterogeneity across studies was tested using the *I*^2^ statistic, a quantitative measure of inconsistency across studies. Studies with an *I*^2^ of 25–50% were considered to have low heterogeneity; *I*^2^ of 50–75%: moderate heterogeneity, and *I*^2^ > 75%: high heterogeneity (Borenstein et al., 2017).

## Results

### Methodological Quality

The selected studies were analyzed through TESTEX, and then, two articles were excluded for having poor quality (≤6 points). Two articles obtained 7 points. Three articles obtained 8 points, and five obtained 9 points. Only two articles were deemed of high quality, and each obtained 10 points (Table 1).

**TABLE 1:** Quality analysis results according to TESTEX.

Autor (year)	A	B	C	D	E	F*	F**	F***	G	H^#^	H^##^	I	J	K	M	Sum
Artioli et al., 2010a	Yes	No	Yes	Yes	No	Yes	Yes	No	No	Yes	Yes	Yes	No	No	No	**8**
Koral and Dosseville, 2009	No	No	Yes	Yes	No	Yes	No	No	No	Yes	Yes	Yes	Yes	No	No	**7**
Barbas et al., 2011 ^@^	No	No	Yes	No	No	Yes	Yes	Yes	No	No	Yes	Yes	No	No	No	**6**
Finaud et al., 2006	Yes	Yes	Yes	Yes	No	Yes	Yes	Yes	No	Yes	Yes	Yes	No	No	No	**10**
Finaud et al., 2006 ^$^	Yes	Yes	Yes	Yes	No	Yes	No	Yes	No	Yes	Yes	Yes	No	No	No	**9**
Finn et al., 2004	Yes	No	No	Yes	No	Yes	Yes	Yes	No	Yes	Yes	Yes	No	No	Yes	**9**
Hall and Lane, 2001	Yes	No	Yes	Yes	No	Yes	No	Yes	No	Yes	Yes	Yes	Yes	No	No	**9**
Lopes-Silva et al., 2014	No	Yes	Yes	Yes	Yes	No	Yes	Yes	No	Yes	Yes	Yes	No	No	No	**9**
Motevalli et al., 2015	Yes	Yes	No	Yes	No	Yes	No	No	No	Yes	Yes	Yes	No	No	No	**7**
Timpmann et al., 2012	Yes	Yes	Yes	Yes	Yes	Yes	No	No	No	Yes	Yes	Yes	Yes	No	No	**10**
Reale et al., 2017	No	Yes	Yes	Yes	No	Yes	Yes	Yes	No	Yes	Yes	No	No	No	Yes	**9**
Rhyu and Cho, 2014	Yes	Yes	No	Yes	No	Yes	Yes	No	No	Yes	Yes	Yes	No	No	No	**8**
Tarnopolsky et al., 1996 ^@^	Yes	Yes	Yes	Yes	No	No	No	Yes	No	Yes	No	No	No	No	No	**6**
Zubac et al., 2020	Yes	No	Yes	Yes	Yes	No	No	No	No	Yes	Yes	Yes	Yes	No	No	**8**

Yes = 1 point; No = 0 points; A Eligibility Criteria Specified; B Randomly Allocated Participants; C Allocation concealment; D Groups similar at baseline; E Blinding of assessor; F Outcome measures assessed in 85% of patients; G Intention-to-treat analysis; H Between-group statistical comparisons reported; I Point measures and measures of variability reported; J Activity monitoring in control groups; K Relative exercise intensity remained constant; M Exercise volume and energy expenditure; * > 85% adherence; **notified adverse effects; ***notified% assistance; ^#^for principal outcomes; ^##^for secondary outcomes. In yellow excluded for points, ^$^excluded for duplicate data, ^@^excluded for incomplete data.

Table 2 summarizes the included studies describing the RWL method and performance-related aspects.

**TABLE 2:** Studies included with RWL and performance-related aspects (i.e., power, strength, fatigue).

Reference	Intervention weight	Intervention performance	Weight loss	Days	Outcome
Reale et al., 2017	One day with fluid restriction	Lower body power and repeated sprint ability (RSA) tests, countermovement jump (CMJ)	−3.20%	6	= Increase*
Finn et al., 2004	Dehydration	Intermittent sprint work and a mood inventory	−4.60%	4	= Increase*
Motevalli et al., 2015	Gradual weight loss	Serum myostatin and follistain	−4%	10	= Increase
	Rapid weight loss	Serum myostatin and follistain	−4%	2	Decrease*
Timpmann et al., 2012	Energy and fluid restriction	Upper body intermittent sprint performance (UBISP) (fatigue)	−5%	3	Decrease*
Lopes-Silva et al., 2014	Lose weight using the habitual methods for lose weight	Special Judo fitness test (SJFT)	−5%	5	= Increase
Artioli et al., 2010a	Reducing fluid intake, exercising with plastic suits, and/or exercising in heated environments (−5%), 4 h to re-feed and rehydrate.	Wingate performance and judo specific exercise.	−5%	5	= Increase
Koral and Dosseville, 2009	6 days before test, dehydration (sweating through exercise in plastic suits)	Squat jump, countermovement jump, mean power, tokui-waza during 30 s	−3.90%	6	= Increase*
Finaud et al., 2006	Restriction fluid and food for 1 week	Psychological factors and grip strength	−5%	7	Decrease*
Hall and Lane, 2001	Restriction fluid and food for 1 week	Circuit training task	−5%	7	Decrease
Rhyu and Cho, 2014	Weight loss through 3 weeks of ketogenic diet or non-ketogenic diet both 25% less calories	Peak power, mean power, anaerobic fatigue, grip force	4–5.9%	21	Increase*
Zubac et al., 2020	Restriction fluid and food for 1 week	Fatigue protocol	3%	3	Decrease*

* = statistical significance ≤0.05.

### Meta-Analyses

**FIGURE 2:**
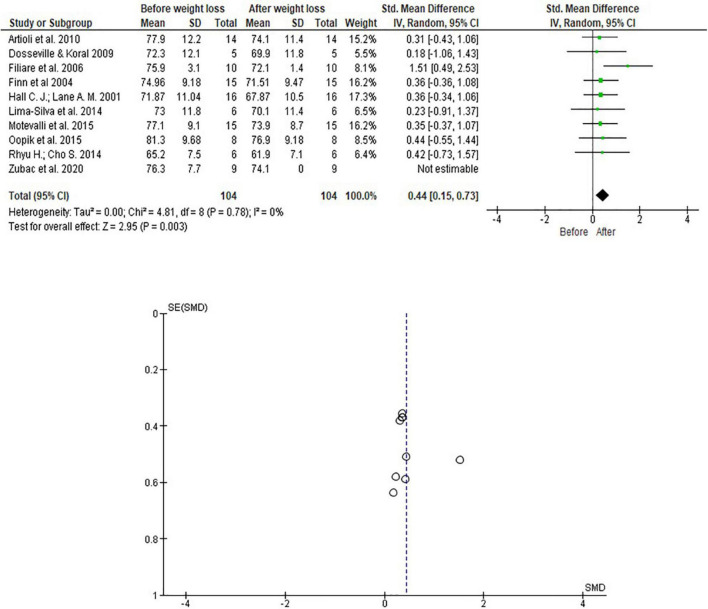
Meta-analysis considering RWL studies with ∼3% of Olympic combat athletes’ body mass.

Figure 2 shows the studies that address RWL in less than 10 days of above 3% of their body weight. This analysis showed a significant difference in weight loss for combat athletes (*p* = 0.003).

Results pictorially represented in Figure 2 indicated a significant and positive impact of RWL in studies analyzed, with a medium effect [0.44 (0.15,0.73)]. The analysis did not detect heterogeneity between studies included in the meta-analysis.

**FIGURE 3:**
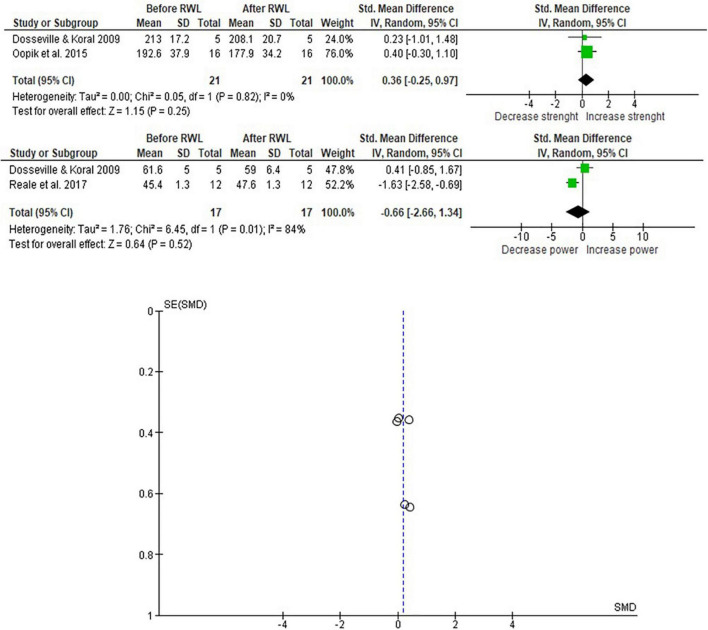
Meta-analysis considering RWL studies with ∼5% of Olympic combat athletes’ body mass.

Figure 3 shows the studies in which the athletes lose less than 5% of their body weight in less than 10 days. The mean power analysis was considered for strength evaluation and the countermovement jump test for power. RWL did not affect performance (*p* = 0.52).

Results in Figure 3 did not demonstrate a significant impact of RWL in strength and power evaluations, with a small effect [0.36 (−0.25,0.97)] and negative medium effect [0.66 (−2.66,1.34)]. The analysis detected a high heterogeneity between countermovement jump test results associated with RWL.

**FIGURE 4:**
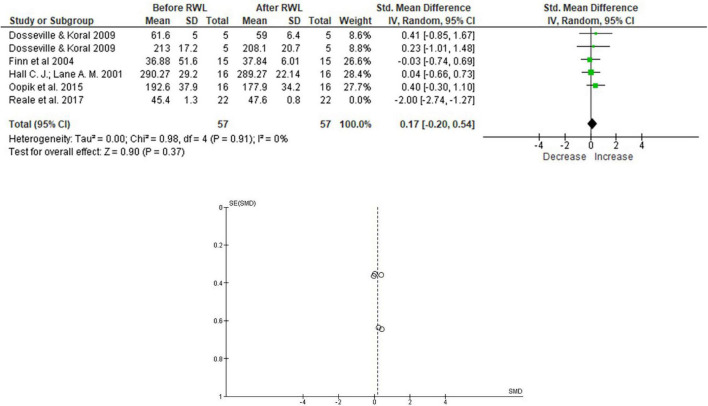
Meta-analysis of the intermittent sprint work and power considering RWL studies with Olympic combat athletes.

Figure 4 demonstrates the analysis of the intermittent sprint work and power; no significant difference was seen concerning performance, as shown in Figure 4.

Results in Figure 4 did not demonstrate a significant impact of RWL on strength and power evaluations, with a small effect [0.17 (−0.20,0.54)]. The analysis did not detect heterogeneity between studies included in the meta-analysis.

## Discussion

The RWL is a traditional practice in combat sports, but it can be harmful to health and detrimentally affect athletic performance. Despite the importance of this topic for the scientific community, coaches, and athletes, we are not aware of a meta-analysis that has analyzed the effect of RWL on the performance of fighters. Our meta-analysis has shown a significant effect size for 5% lost weight, while muscle power and strength were not affected during RWL. The analysis showed a decrease in perceived fatigue when the athletes lost weight, and this was, but, unrelated to strength and muscle power.

Studies included in this meta-analysis investigated the impact of RWL of a moderate range, typically of ∼3–5% of the body mass, and its effect on performance outcomes. Cases of more significant weight cuts are recognized, although, and are a reality of certain sports, such as Mixed Martial Arts (MMA) (Barley et al., 2018). In British athletes preparing for an MMA event, Matthews and Nicholas (2017) observed that the loss in the pre-competition week was close to 8% of the body mass. However, 32 h after weighing, the athlete’s recovery was about 11.7%. According to Coswig et al. (2019), the ability to regain weight is associated with competitive success in MMA. These more significant magnitude weight cuts should be analyzed to assess the effect on performance measures following a standard recovery period (Matthews and Nicholas, 2017; Coswig et al., 2019). A significant number of athletes can lose more than 5% of their body mass, which could be potentially dangerous for the participant’s health and safety and detrimental for physical performance (Barley et al., 2018; Coswig et al., 2019).

The RWL methods are often seen in combat sports, including pharmacological methods such as the use of diuretics and laxatives; moreover, it seems that weight loss methods such as sauna or training with plastic clothes are conventional to dehydrate as well (Brito et al., 2012). In this review, most of the included studies performed dehydration to rapidly lose weight (Oöpik et al., 1996; Motevalli et al., 2015; Reale et al., 2017). Athletes are believed to have more chances of winning if practicing RWL, as shown by Horswill et al. (1994), who suggested that weight-loss athletes might have a greater likelihood of success. This seems to be interesting since no effects were seen after weight loss (3–5% of the body mass) in a short period (3–6 days) in terms of strength tests; furthermore, this corroborates what was presented by Finn et al. (2004) and Koral and Dosseville (2009) who performed potency analyses and found that potency did not decrease after the loss of approximately 4% of body weight through dehydration. However, in terms of health, it is important to highlight that RWL could be linked with relative energy deficiency and impaired physiological functions (i.e., metabolic rate, body composition, cardiorespiratory, menstrual function, bone health, and immunity) and detrimental responsiveness to training and competition (i.e., training load, injury, and recovery) (Roklicer et al., 2020; Miles-Chan and Isacco, 2021) and even kidney damage (Lakicevic et al., 2020). More chronic health studies are needed in the arena of combat sports.

Regarding our performance in the meta-analysis, the study by Reale et al. (2017) was not taken into account because it did not present the mean weight and SD after weight loss; thus, even though it was included based on the selection criteria, its results were excluded due to lack of data. However, other researchers (Barbas et al., 2011) analyzed the handgrip test with wrestlers after losing approximately 6% of body weight, which showed a significant decrease in strength. Finaud et al. (2006) concluded that RWL (−5%) could cause negative psychological (tension, vigor, and fatigue) and physical impacts. Using another measurement methodology, Hall and Lane (2001) elaborated a boxing training circuit where they measured performance, the number of hits, and mood parameters such as anger, confusion, depression, fatigue, tension, and vigor after the rapid loss of 5% of the initial weight. They concluded that RWL impairs performance and negatively affects anger, fatigue, tension, and vigor. However, in another study, Rhyu and Cho (2014) performed a more extended intervention with 21 days of an energy-restricted ketogenic and non-ketogenic diet approach to lose 4–6% of the weight, and they could not see a significant difference in terms of the handgrip test.

Therefore, it seems that the processes of RWL of up to 5% of body weight can be beneficial to athletes, as they do not negatively influence their performance, taking into account the aspects of power and strength, as already suggested by Mendes et al. (2013); while Artioli et al. (2010a) emphasized that the determining factor for decreased performance would be hydration or food after weighing and before the fight. We emphasized the importance of conducting new studies and adopting more robust methodologies to assess the psychological effects of the process of RWL in combat athletes as the evaluation parameters and methodologies retained in this review are highly different and heterogeneous, despite being mentioned by some studies (Hall and Lane, 2001; Finaud et al., 2006; Franchini et al., 2012). This is reinforced by a previous study by Matthews et al. (2019), which indicated that the magnitude of RWL appears to be influenced by the type of sport, competition structure, and recovery duration permitted. A cause for concern is the lack of objective data quantifying the magnitude of RWL. There is insufficient evidence to substantiate the use of RWG as a proxy for RWL, and a few data are available in women (i.e., Amatori et al., 2020; Cannataro et al., 2020; Janiszewska and Przybylowicz, 2020) and younger athletes (i.e., Artioli et al., 2010a; Kazemi et al., 2011; Berkovich et al., 2016; Drid et al., 2021), suggesting the need for further studies specifically targeting these groups.

Regarding the classification of articles, we observed some weaknesses among the selected ones about the criteria addressed for classification, such as the researcher blinding. In this type of study, it may be that the researcher blinding does not interfere, as one of the primary outcomes in weight loss, and this has been achieved. Another observation is about the intention to treat, as they are acute studies, they only verified weight loss and how this can influence performance; since they are athletes, prior knowledge and discussions on this topic will bring new information to the researchers and thus could lead to better post-research and post-weight loss interventions in practice. Other items that compromised the quality of the studies according to this evaluation method were the monitoring of the control group and the criteria related to exercise intensity, volume, and energy expenditure. As these are studies in which the intervention is often its control, many did not have this item; as for the other items, they are field studies where athletes were often in the middle of training routines, and they could not go to a research center, and interventions (weight loss) were carried out on consecutive days, which makes it challenging to obtain the analyses of exercise intensity, volume, and energy expenditure, which would be performed in the laboratory or would require greater availability of the athlete.

In addition, strength and power performance in combat sports are associated with attacks and defensive actions during tournaments, in which maximal intensity actions are sustained until 10–30 s (Reaburn and Dascombe, 2008). The substrate used for anaerobic actions during combat sports due to a higher activity intensity is carbohydrate dependent once the activity crests over approximately 60–85% of an athlete’s VO_2*max*_ (Franchini et al., 2011). This energy system consumes stored glycogen within the muscles and the liver as a source for rapid energy delivery during the higher intensity fights. Glycogen is then converted to glucose and then shuttled to the bloodstream to fuel ATP production. However, during anaerobic processes lasting even shorter in duration, approximately 5–6 s, the creatine phosphate cycle becomes the preferred option to produce ATP within that period (Powers and Howley, 2017). The creatine phosphate molecule transfers its phosphate group to adenosine diphosphate (ADP), rapidly transforming into ATP (Powers and Howley, 2017). Due to the limited reserve amount of creatine phosphate stored within the body, the supply of ATP from this process can only last for a short duration. Research has been carried out to examine dehydration’s effect on this energy system’s performance. However, no meta-analysis has observed the effect size of the 3–5% body mass in RWL suggested in the literature on anaerobic performance. This information would be essential as it could assist coaches in planning activities during the week prior to the competitive tournament.

## Conclusion

This investigation based on a systematic search and appraisal of the literature found that RWL of up to 5% of the body mass in less than 7 days does not seem to influence performance, considering the strength and the power. However, this review, currently, is based on a few studies in the literature that have evaluated weight loss and analyzed performance in combat sports athletes, requiring further studies to elucidate the best methods, time, and percentage of weight loss that do not negatively influence performance.

## Data Availability Statement

The original contributions presented in the study are included in the article/supplementary material, further inquiries can be directed to the corresponding author.

## Author Contributions

All authors listed have made a substantial, direct, and intellectual contribution to the work, and approved it for publication.

## Conflict of Interest

The authors declare that the research was conducted in the absence of any commercial or financial relationships that could be construed as a potential conflict of interest.

## Publisher’s Note

All claims expressed in this article are solely those of the authors and do not necessarily represent those of their affiliated organizations, or those of the publisher, the editors and the reviewers. Any product that may be evaluated in this article, or claim that may be made by its manufacturer, is not guaranteed or endorsed by the publisher.

## Footnotes

1
https://training.cochrane.org/online-learning/core-software-cochrane-reviews/revman
